# Benzene-1,3-diol–1,4-diaza­bicyclo­[2.2.2]octane (1/1)

**DOI:** 10.1107/S1600536810030199

**Published:** 2010-07-31

**Authors:** Hadi D. Arman, Edward R. T. Tiekink

**Affiliations:** a Department of Chemistry, The University of Texas at San Antonio, One UTSA Circle, San Antonio, Texas 78249-0698, USA; b Department of Chemistry, University of Malaya, 50603 Kuala Lumpur, Malaysia

## Abstract

There are two independent but virtually identical mol­ecules of each component in the asymmetric unit of the title 1:1 adduct, C_6_H_12_N_2_·C_6_H_6_O_2_. In the crystal, the constituents are connected into a supra­molecular chain along the *b* axis by O—H⋯N hydrogen bonds. Weak C—H⋯O bonds cross-link the chains.

## Related literature

For related studies on co-crystal/adduct formation, see: Broker & Tiekink (2007 ▶); Broker *et al.* (2008 ▶); Arman *et al.* (2010 ▶).
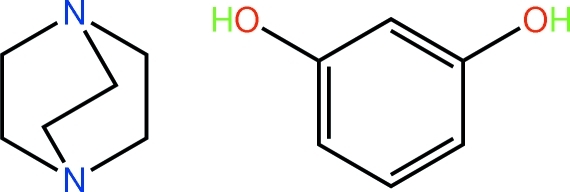


## Experimental

### 

#### Crystal data

C_6_H_12_N_2_·C_6_H_6_O_2_
*M* *_r_* = 222.28
Monoclinic, 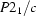

*a* = 9.3620 (19) Å
*b* = 23.645 (5) Å
*c* = 11.072 (2) Å
β = 112.64 (3)°
*V* = 2262.1 (8) Å^3^
*Z* = 8
Mo *K*α radiation
μ = 0.09 mm^−1^
*T* = 98 K
0.40 × 0.25 × 0.07 mm

#### Data collection

Rigaku AFC12/SATURN724 diffractometer
Absorption correction: multi-scan (*ABSCOR*; Higashi, 1995 ▶) *T*_min_ = 0.423, *T*_max_ = 1.000
11918 measured reflections
3973 independent reflections
3355 reflections with *I* > 2σ(*I*)
*R*_int_ = 0.049

#### Refinement

*R*[*F* ^2^ > 2σ(*F* ^2^)] = 0.064
*wR*(*F* ^2^) = 0.157
*S* = 1.00
3973 reflections
301 parameters
4 restraints
H atoms treated by a mixture of independent and constrained refinement
Δρ_max_ = 0.27 e Å^−3^
Δρ_min_ = −0.24 e Å^−3^

### 

Data collection: *CrystalClear* (Molecular Structure Corporation & Rigaku, 2005 ▶); cell refinement: *CrystalClear*; data reduction: *CrystalClear*; program(s) used to solve structure: *SHELXS97* (Sheldrick, 2008 ▶); program(s) used to refine structure: *SHELXL97* (Sheldrick, 2008 ▶); molecular graphics: *ORTEP-3* (Farrugia, 1997 ▶) and *DIAMOND* (Brandenburg, 2006 ▶); software used to prepare material for publication: *publCIF* (Westrip, 2010 ▶).

## Supplementary Material

Crystal structure: contains datablocks global, I. DOI: 10.1107/S1600536810030199/hb5588sup1.cif

Structure factors: contains datablocks I. DOI: 10.1107/S1600536810030199/hb5588Isup2.hkl

Additional supplementary materials:  crystallographic information; 3D view; checkCIF report

## Figures and Tables

**Table 1:** Hydrogen-bond geometry (Å, °)

*D*—H⋯*A*	*D*—H	H⋯*A*	*D*⋯*A*	*D*—H⋯*A*
O1—H1*O*⋯N1^i^	0.85 (2)	1.81 (2)	2.639 (3)	167 (3)
O2—H2*O*⋯N2	0.85 (3)	1.84 (2)	2.670 (3)	169 (3)
O3—H3*O*⋯N3^ii^	0.85 (2)	1.88 (2)	2.718 (3)	171 (2)
O4—H4*O*⋯N4	0.85 (2)	1.93 (2)	2.763 (3)	169 (3)
C23—H23⋯O1^iii^	0.95	2.55	3.330 (3)	139

Symmetry codes: (i) 
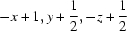
; (ii) 
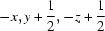
; (iii) 
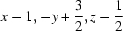
.

**Fig. 1.**
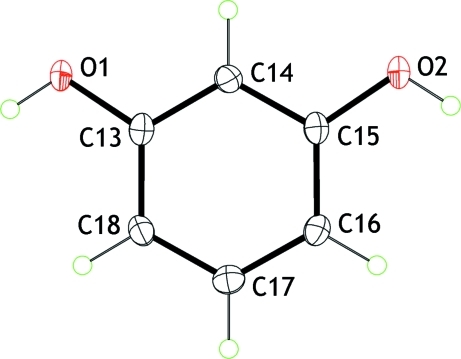
Molecular structure of the first independent benzene-1,3-diol molecule in (I) showing displacement ellipsoids at the 50% probability level.

**Fig. 2.**
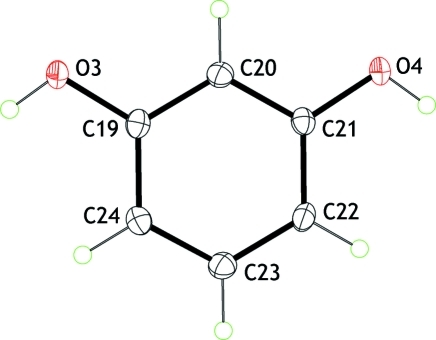
Molecular structure of the second independent benzene-1,3-diol molecule in (I) showing displacement ellipsoids at the 50% probability level.

**Fig. 3.**
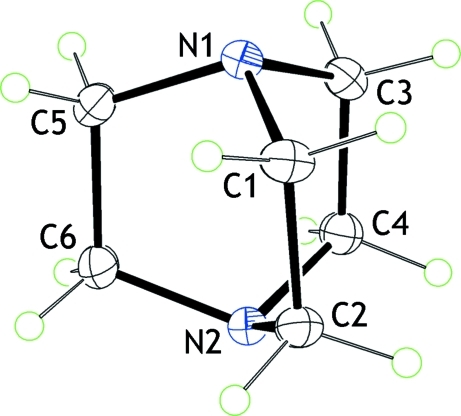
Molecular structure of the first independent 1,4-diazabicyclo[2.2.2]octane molecule in (I) showing displacement ellipsoids at the 50% probability level.

**Fig. 4.**
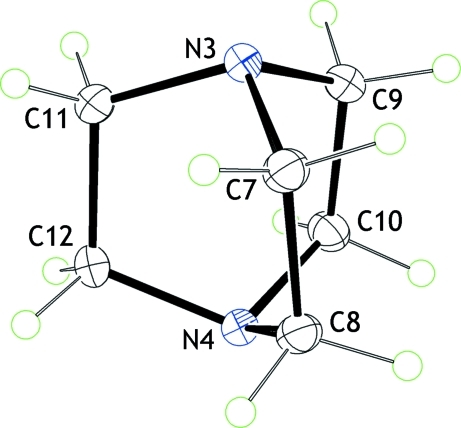
Molecular structure of the second independent 1,4-diazabicyclo[2.2.2]octane molecule in (I) showing displacement ellipsoids at the 50% probability level.

**Fig. 5.**
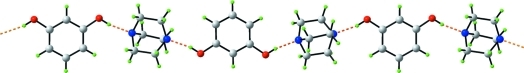
Supramolecular chain along the b axis in (I) mediated by O–H···N hydrogen bonding (orange dashed lines).

**Fig. 6.**
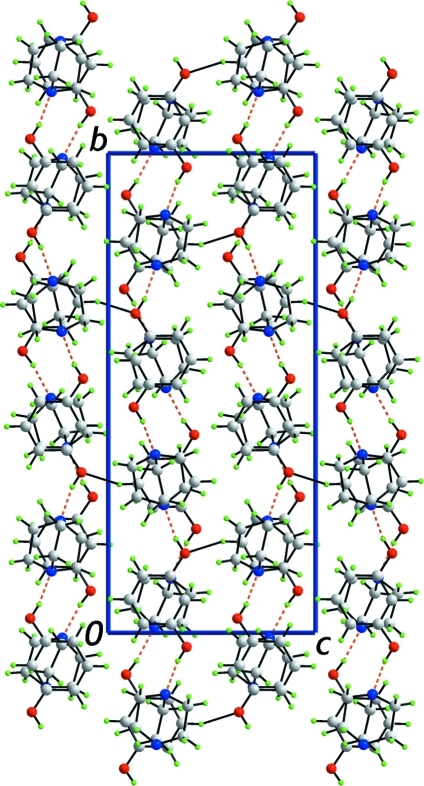
View in projection down the a axis of the unit-cell contents of (I). The O–H···N hydrogen bonding and C–H···O contacts are shown as orange and blue dashed lines, respectively.

## supplementary crystallographic information

### Comment

As a part of on-going studies into co-crystallization experiments with N-containing molecules (Broker & Tiekink, 2007; Broker *et al.*, 2008; Arman *et al.* 2010), the co-crystallization of benzene-1,3-diol and 1,4-diazabicyclo[2.2.2]octane (dabco) was investigated, leading to the isolation of the 1:1 co-crystal, (I).

The crystallographic asymmetric unit of (I) comprises two independent benzene-1,3-diol molecules, Figs 1 and 2, and two independent dabco molecules, Figs 3 and 4. The molecules associate *via* O–H···N hydrogen bonds with each benzene-1,3-diol molecule bridging two independent dabco molecules. This results in the formation of a supramolecular chain along the *b* axis, Fig. 5 and Table 1. Chains are consolidated in the crystal structure by C–H···O contacts, Fig. 6 and Table 1.

### Experimental

Colourless prisms of (I) were isolated from the 1/1 co-crystallization of 1,4-diazabicyclo[2.2.2]octane (Sigma-Aldrich, 0.18 mmol) and benzene-1,3-diol (ACROS, 0.18 mmol) in acetone/ethanol solution, m. pt. 513–517 K

### Refinement

The C-bound H-atoms were placed in calculated positions (C–H 0.95–0.99 Å) and were included in the refinement in the riding model approximation with *U*_iso_(H) set to 1.2*U*_eq_(C). The O-bound H-atoms were located in a difference Fourier map and were refined with a distance restraint of O–H 0.84±0.01 Å, and with *U*_iso_(H) = 1.5*U*_eq_(O).

### Crystal data

**Table:**

C_6_H_12_N_2_·C_6_H_6_O_2_	*F*(000) = 960
*M**_r_* = 222.28	*D*_x_ = 1.305 Mg m^−^^3^
Monoclinic, *P*2_1_/*c*	Mo *K*α radiation, λ = 0.71073 Å
Hall symbol: -P 2ybc	Cell parameters from 10564 reflections
*a* = 9.3620 (19) Å	θ = 2.0–40.2°
*b* = 23.645 (5) Å	µ = 0.09 mm^−^^1^
*c* = 11.072 (2) Å	*T* = 98 K
β = 112.64 (3)°	Prism, colourless
*V* = 2262.1 (8) Å^3^	0.40 × 0.25 × 0.07 mm
*Z* = 8	

### Data collection

**Table:**

Rigaku AFC12K/SATURN724 diffractometer	3973 independent reflections
Radiation source: fine-focus sealed tube	3355 reflections with *I* > 2σ(*I*)
graphite	*R*_int_ = 0.049
Detector resolution: 28.5714 pixels mm^-1^	θ_max_ = 25.0°, θ_min_ = 2.2°
ω scans	*h* = −8→11
Absorption correction: multi-scan (*ABSCOR*; Higashi, 1995)	*k* = −25→28
*T*_min_ = 0.423, *T*_max_ = 1.000	*l* = −13→13
11918 measured reflections	

### Refinement

**Table:**

Refinement on *F*^2^	Primary atom site location: structure-invariant direct methods
Least-squares matrix: full	Secondary atom site location: difference Fourier map
*R*[*F*^2^ > 2σ(*F*^2^)] = 0.064	Hydrogen site location: inferred from neighbouring sites
*wR*(*F*^2^) = 0.157	H atoms treated by a mixture of independent and constrained refinement
*S* = 1.00	*w* = 1/[σ^2^(*F*_o_^2^) + (0.0661*P*)^2^ + 2.685*P*] where *P* = (*F*_o_^2^ + 2*F*_c_^2^)/3
3973 reflections	(Δ/σ)_max_ < 0.001
301 parameters	Δρ_max_ = 0.27 e Å^−^^3^
4 restraints	Δρ_min_ = −0.24 e Å^−^^3^

### Special details

**Table:**

Geometry. All s.u.'s (except the s.u. in the dihedral angle between two l.s. planes) are estimated using the full covariance matrix. The cell s.u.'s are taken into account individually in the estimation of s.u.'s in distances, angles and torsion angles; correlations between s.u.'s in cell parameters are only used when they are defined by crystal symmetry. An approximate (isotropic) treatment of cell s.u.'s is used for estimating s.u.'s involving l.s. planes.
Refinement. Refinement of *F*^2^ against ALL reflections. The weighted *R*-factor *wR* and goodness of fit *S* are based on *F*^2^, conventional *R*-factors *R* are based on *F*, with *F* set to zero for negative *F*^2^. The threshold expression of *F*^2^ > 2σ(*F*^2^) is used only for calculating *R*-factors(gt) *etc*. and is not relevant to the choice of reflections for refinement. *R*-factors based on *F*^2^ are statistically about twice as large as those based on *F*, and *R*- factors based on ALL data will be even larger.

### Fractional atomic coordinates and isotropic or equivalent isotropic displacement parameters (Å^2^)

**Table:**

	*x*	*y*	*z*	*U*_iso_*/*U*_eq_	
O1	0.6822 (2)	1.16554 (7)	0.36932 (18)	0.0301 (5)	
H1O	0.648 (4)	1.1955 (8)	0.326 (3)	0.045*	
O2	0.6063 (2)	0.97031 (7)	0.37749 (18)	0.0314 (5)	
H2O	0.562 (4)	0.9413 (9)	0.335 (3)	0.047*	
O3	0.0808 (2)	0.41271 (7)	0.41406 (17)	0.0263 (4)	
H3O	0.051 (4)	0.4413 (9)	0.364 (2)	0.039*	
O4	0.1800 (2)	0.21654 (7)	0.42386 (17)	0.0266 (4)	
H4O	0.149 (4)	0.1873 (8)	0.377 (3)	0.040*	
N1	0.4071 (3)	0.76680 (8)	0.2317 (2)	0.0228 (5)	
N2	0.4791 (3)	0.87151 (8)	0.2746 (2)	0.0219 (5)	
N3	0.0069 (3)	0.01097 (8)	0.22198 (19)	0.0205 (5)	
N4	0.0848 (3)	0.11358 (8)	0.30117 (19)	0.0202 (5)	
C1	0.2920 (3)	0.80228 (10)	0.1308 (3)	0.0269 (6)	
H1A	0.1892	0.7976	0.1356	0.032*	
H1B	0.2838	0.7901	0.0428	0.032*	
C2	0.3406 (3)	0.86513 (10)	0.1519 (2)	0.0245 (6)	
H2A	0.3639	0.8789	0.0770	0.029*	
H2B	0.2546	0.8881	0.1568	0.029*	
C3	0.5626 (3)	0.77703 (10)	0.2309 (3)	0.0262 (6)	
H3A	0.5622	0.7678	0.1435	0.031*	
H3B	0.6391	0.7522	0.2961	0.031*	
C4	0.6094 (3)	0.83966 (10)	0.2637 (3)	0.0269 (6)	
H4A	0.7005	0.8420	0.3473	0.032*	
H4B	0.6378	0.8564	0.1941	0.032*	
C5	0.4101 (4)	0.78343 (10)	0.3614 (3)	0.0275 (6)	
H5A	0.4911	0.7617	0.4307	0.033*	
H5B	0.3090	0.7747	0.3663	0.033*	
C6	0.4438 (4)	0.84720 (10)	0.3833 (2)	0.0274 (6)	
H6A	0.3528	0.8667	0.3887	0.033*	
H6B	0.5329	0.8532	0.4671	0.033*	
C7	−0.1050 (3)	0.05173 (10)	0.1344 (2)	0.0240 (6)	
H7A	−0.2073	0.0464	0.1396	0.029*	
H7B	−0.1166	0.0448	0.0429	0.029*	
C8	−0.0488 (3)	0.11313 (10)	0.1737 (2)	0.0240 (6)	
H8A	−0.0181	0.1303	0.1057	0.029*	
H8B	−0.1342	0.1360	0.1803	0.029*	
C9	0.1632 (3)	0.02439 (10)	0.2251 (3)	0.0241 (6)	
H9A	0.1609	0.0237	0.1350	0.029*	
H9B	0.2378	−0.0046	0.2770	0.029*	
C10	0.2163 (3)	0.08326 (10)	0.2863 (3)	0.0249 (6)	
H10A	0.3018	0.0790	0.3729	0.030*	
H10B	0.2552	0.1055	0.2296	0.030*	
C11	0.0090 (3)	0.01896 (10)	0.3555 (2)	0.0239 (6)	
H11A	0.0908	−0.0050	0.4182	0.029*	
H11B	−0.0917	0.0072	0.3572	0.029*	
C12	0.0401 (3)	0.08194 (10)	0.3972 (2)	0.0227 (6)	
H12A	−0.0543	0.0989	0.4019	0.027*	
H12B	0.1241	0.0844	0.4850	0.027*	
C13	0.6158 (3)	1.11889 (10)	0.2989 (3)	0.0227 (6)	
C14	0.6381 (3)	1.06812 (10)	0.3673 (2)	0.0237 (6)	
H14	0.6957	1.0678	0.4591	0.028*	
C15	0.5772 (3)	1.01792 (10)	0.3030 (2)	0.0221 (5)	
C16	0.4901 (3)	1.01835 (10)	0.1682 (2)	0.0234 (6)	
H16	0.4480	0.9843	0.1231	0.028*	
C17	0.4663 (3)	1.06950 (10)	0.1012 (2)	0.0245 (6)	
H17	0.4067	1.0700	0.0097	0.029*	
C18	0.5274 (3)	1.11975 (10)	0.1644 (2)	0.0238 (6)	
H18	0.5095	1.1543	0.1171	0.029*	
C19	0.0690 (3)	0.36384 (10)	0.3458 (2)	0.0207 (5)	
C20	0.1297 (3)	0.31467 (10)	0.4162 (2)	0.0219 (5)	
H20	0.1791	0.3161	0.5089	0.026*	
C21	0.1181 (3)	0.26331 (10)	0.3511 (2)	0.0205 (5)	
C22	0.0461 (3)	0.26154 (10)	0.2147 (2)	0.0234 (6)	
H22	0.0377	0.2267	0.1696	0.028*	
C23	−0.0129 (3)	0.31076 (10)	0.1454 (2)	0.0246 (6)	
H23	−0.0601	0.3094	0.0526	0.030*	
C24	−0.0042 (3)	0.36198 (10)	0.2093 (2)	0.0235 (6)	
H24	−0.0473	0.3953	0.1611	0.028*	

### Atomic displacement parameters (Å^2^)

**Table:**

	*U*^11^	*U*^22^	*U*^33^	*U*^12^	*U*^13^	*U*^23^
O1	0.0397 (12)	0.0137 (9)	0.0289 (10)	−0.0014 (8)	0.0046 (9)	−0.0009 (7)
O2	0.0439 (13)	0.0143 (9)	0.0301 (10)	−0.0031 (8)	0.0077 (10)	0.0008 (7)
O3	0.0398 (12)	0.0156 (8)	0.0241 (9)	0.0037 (8)	0.0132 (9)	0.0001 (7)
O4	0.0385 (12)	0.0141 (8)	0.0251 (9)	0.0026 (8)	0.0098 (9)	0.0011 (7)
N1	0.0286 (13)	0.0180 (10)	0.0238 (11)	0.0002 (9)	0.0124 (10)	−0.0012 (8)
N2	0.0272 (13)	0.0181 (10)	0.0226 (11)	−0.0002 (9)	0.0121 (10)	0.0000 (8)
N3	0.0256 (12)	0.0182 (10)	0.0204 (10)	0.0006 (8)	0.0118 (9)	−0.0003 (8)
N4	0.0245 (12)	0.0172 (10)	0.0211 (10)	0.0004 (8)	0.0114 (9)	0.0006 (8)
C1	0.0279 (15)	0.0211 (13)	0.0276 (13)	0.0016 (11)	0.0062 (12)	−0.0009 (10)
C2	0.0265 (15)	0.0200 (12)	0.0261 (13)	0.0020 (10)	0.0092 (12)	0.0016 (10)
C3	0.0283 (15)	0.0238 (13)	0.0289 (13)	0.0026 (11)	0.0137 (12)	−0.0024 (10)
C4	0.0273 (15)	0.0246 (13)	0.0326 (14)	−0.0010 (11)	0.0158 (12)	−0.0009 (11)
C5	0.0372 (17)	0.0230 (13)	0.0262 (13)	−0.0039 (11)	0.0164 (13)	0.0014 (10)
C6	0.0378 (17)	0.0245 (13)	0.0239 (13)	−0.0037 (11)	0.0162 (13)	−0.0026 (10)
C7	0.0278 (15)	0.0211 (12)	0.0225 (12)	−0.0004 (10)	0.0090 (12)	0.0010 (10)
C8	0.0309 (16)	0.0184 (12)	0.0218 (12)	0.0016 (11)	0.0091 (12)	0.0018 (10)
C9	0.0290 (15)	0.0199 (12)	0.0274 (13)	0.0017 (11)	0.0152 (12)	−0.0017 (10)
C10	0.0248 (15)	0.0229 (12)	0.0304 (13)	−0.0024 (11)	0.0145 (12)	−0.0044 (10)
C11	0.0333 (16)	0.0190 (12)	0.0225 (12)	0.0013 (11)	0.0142 (12)	0.0026 (10)
C12	0.0281 (15)	0.0237 (12)	0.0205 (12)	0.0008 (10)	0.0138 (12)	−0.0004 (10)
C13	0.0221 (14)	0.0177 (12)	0.0302 (13)	0.0000 (10)	0.0121 (12)	−0.0013 (10)
C14	0.0249 (14)	0.0218 (12)	0.0223 (12)	0.0035 (10)	0.0069 (11)	0.0006 (10)
C15	0.0231 (14)	0.0174 (12)	0.0288 (13)	0.0009 (10)	0.0134 (12)	0.0005 (10)
C16	0.0263 (15)	0.0199 (12)	0.0262 (13)	−0.0015 (10)	0.0126 (12)	−0.0040 (10)
C17	0.0246 (15)	0.0294 (13)	0.0221 (12)	0.0010 (11)	0.0120 (11)	−0.0021 (10)
C18	0.0286 (15)	0.0193 (12)	0.0258 (13)	0.0026 (10)	0.0127 (12)	0.0037 (10)
C19	0.0199 (14)	0.0183 (12)	0.0265 (13)	−0.0023 (10)	0.0118 (11)	−0.0011 (10)
C20	0.0249 (14)	0.0228 (12)	0.0205 (12)	−0.0013 (10)	0.0116 (11)	−0.0001 (10)
C21	0.0231 (14)	0.0183 (12)	0.0239 (12)	−0.0002 (10)	0.0132 (11)	0.0016 (9)
C22	0.0285 (15)	0.0167 (12)	0.0265 (13)	−0.0028 (10)	0.0123 (12)	−0.0037 (10)
C23	0.0287 (15)	0.0252 (13)	0.0217 (12)	−0.0024 (11)	0.0117 (11)	−0.0003 (10)
C24	0.0253 (15)	0.0210 (12)	0.0253 (13)	0.0015 (10)	0.0110 (12)	0.0032 (10)

### Geometric parameters (Å, °)

**Table:**

O1—C13	1.356 (3)	C7—C8	1.548 (3)
O1—H1O	0.85 (2)	C7—H7A	0.9900
O2—C15	1.360 (3)	C7—H7B	0.9900
O2—H2O	0.85 (3)	C8—H8A	0.9900
O3—C19	1.362 (3)	C8—H8B	0.9900
O3—H3O	0.85 (2)	C9—C10	1.544 (3)
O4—C21	1.360 (3)	C9—H9A	0.9900
O4—H4O	0.85 (2)	C9—H9B	0.9900
N1—C1	1.479 (3)	C10—H10A	0.9900
N1—C3	1.479 (4)	C10—H10B	0.9900
N1—C5	1.479 (3)	C11—C12	1.553 (3)
N2—C4	1.477 (3)	C11—H11A	0.9900
N2—C6	1.482 (3)	C11—H11B	0.9900
N2—C2	1.481 (3)	C12—H12A	0.9900
N3—C7	1.478 (3)	C12—H12B	0.9900
N3—C11	1.483 (3)	C13—C14	1.392 (3)
N3—C9	1.484 (3)	C13—C18	1.397 (4)
N4—C8	1.482 (3)	C14—C15	1.389 (3)
N4—C12	1.486 (3)	C14—H14	0.9500
N4—C10	1.488 (3)	C15—C16	1.398 (4)
C1—C2	1.545 (3)	C16—C17	1.391 (3)
C1—H1A	0.9900	C16—H16	0.9500
C1—H1B	0.9900	C17—C18	1.386 (3)
C2—H2A	0.9900	C17—H17	0.9500
C2—H2B	0.9900	C18—H18	0.9500
C3—C4	1.547 (3)	C19—C20	1.393 (3)
C3—H3A	0.9900	C19—C24	1.399 (3)
C3—H3B	0.9900	C20—C21	1.395 (3)
C4—H4A	0.9900	C20—H20	0.9500
C4—H4B	0.9900	C21—C22	1.397 (3)
C5—C6	1.541 (3)	C22—C23	1.387 (3)
C5—H5A	0.9900	C22—H22	0.9500
C5—H5B	0.9900	C23—C24	1.389 (3)
C6—H6A	0.9900	C23—H23	0.9500
C6—H6B	0.9900	C24—H24	0.9500
			
C13—O1—H1O	111 (2)	C7—C8—H8B	109.6
C15—O2—H2O	113 (2)	H8A—C8—H8B	108.1
C19—O3—H3O	112 (2)	N3—C9—C10	110.6 (2)
C21—O4—H4O	110 (2)	N3—C9—H9A	109.5
C1—N1—C3	109.6 (2)	C10—C9—H9A	109.5
C1—N1—C5	108.6 (2)	N3—C9—H9B	109.5
C3—N1—C5	108.2 (2)	C10—C9—H9B	109.5
C4—N2—C6	108.5 (2)	H9A—C9—H9B	108.1
C4—N2—C2	109.38 (19)	N4—C10—C9	110.0 (2)
C6—N2—C2	108.4 (2)	N4—C10—H10A	109.7
C7—N3—C11	107.77 (19)	C9—C10—H10A	109.7
C7—N3—C9	108.61 (19)	N4—C10—H10B	109.7
C11—N3—C9	108.3 (2)	C9—C10—H10B	109.7
C8—N4—C12	108.1 (2)	H10A—C10—H10B	108.2
C8—N4—C10	108.79 (19)	N3—C11—C12	110.32 (19)
C12—N4—C10	108.05 (19)	N3—C11—H11A	109.6
N1—C1—C2	110.2 (2)	C12—C11—H11A	109.6
N1—C1—H1A	109.6	N3—C11—H11B	109.6
C2—C1—H1A	109.6	C12—C11—H11B	109.6
N1—C1—H1B	109.6	H11A—C11—H11B	108.1
C2—C1—H1B	109.6	N4—C12—C11	110.03 (19)
H1A—C1—H1B	108.1	N4—C12—H12A	109.7
N2—C2—C1	109.9 (2)	C11—C12—H12A	109.7
N2—C2—H2A	109.7	N4—C12—H12B	109.7
C1—C2—H2A	109.7	C11—C12—H12B	109.7
N2—C2—H2B	109.7	H12A—C12—H12B	108.2
C1—C2—H2B	109.7	O1—C13—C14	116.7 (2)
H2A—C2—H2B	108.2	O1—C13—C18	123.6 (2)
N1—C3—C4	110.2 (2)	C14—C13—C18	119.7 (2)
N1—C3—H3A	109.6	C13—C14—C15	120.8 (2)
C4—C3—H3A	109.6	C13—C14—H14	119.6
N1—C3—H3B	109.6	C15—C14—H14	119.6
C4—C3—H3B	109.6	O2—C15—C14	116.7 (2)
H3A—C3—H3B	108.1	O2—C15—C16	123.5 (2)
N2—C4—C3	109.8 (2)	C14—C15—C16	119.8 (2)
N2—C4—H4A	109.7	C17—C16—C15	118.9 (2)
C3—C4—H4A	109.7	C17—C16—H16	120.5
N2—C4—H4B	109.7	C15—C16—H16	120.5
C3—C4—H4B	109.7	C18—C17—C16	121.7 (2)
H4A—C4—H4B	108.2	C18—C17—H17	119.2
N1—C5—C6	109.8 (2)	C16—C17—H17	119.1
N1—C5—H5A	109.7	C17—C18—C13	119.1 (2)
C6—C5—H5A	109.7	C17—C18—H18	120.5
N1—C5—H5B	109.7	C13—C18—H18	120.5
C6—C5—H5B	109.7	O3—C19—C20	118.0 (2)
H5A—C5—H5B	108.2	O3—C19—C24	121.8 (2)
N2—C6—C5	110.4 (2)	C20—C19—C24	120.2 (2)
N2—C6—H6A	109.6	C21—C20—C19	120.2 (2)
C5—C6—H6A	109.6	C21—C20—H20	119.9
N2—C6—H6B	109.6	C19—C20—H20	119.9
C5—C6—H6B	109.6	O4—C21—C20	118.1 (2)
H6A—C6—H6B	108.1	O4—C21—C22	122.3 (2)
N3—C7—C8	110.4 (2)	C20—C21—C22	119.6 (2)
N3—C7—H7A	109.6	C23—C22—C21	119.8 (2)
C8—C7—H7A	109.6	C23—C22—H22	120.1
N3—C7—H7B	109.6	C21—C22—H22	120.1
C8—C7—H7B	109.6	C22—C23—C24	121.1 (2)
H7A—C7—H7B	108.1	C22—C23—H23	119.4
N4—C8—C7	110.20 (19)	C24—C23—H23	119.4
N4—C8—H8A	109.6	C23—C24—C19	119.1 (2)
C7—C8—H8A	109.6	C23—C24—H24	120.5
N4—C8—H8B	109.6	C19—C24—H24	120.5

### Hydrogen-bond geometry (Å, °)

**Table:**

*D*—H···*A*	*D*—H	H···*A*	*D*···*A*	*D*—H···*A*
O1—H1O···N1^i^	0.85 (2)	1.81 (2)	2.639 (3)	167 (3)
O2—H2O···N2	0.85 (3)	1.84 (2)	2.670 (3)	169 (3)
O3—H3O···N3^ii^	0.85 (2)	1.88 (2)	2.718 (3)	171 (2)
O4—H4O···N4	0.85 (2)	1.93 (2)	2.763 (3)	169 (3)
C23—H23···O1^iii^	0.95	2.55	3.330 (3)	139

Symmetry codes: (i) −*x*+1, *y*+1/2, −*z*+1/2;  (ii) −*x*, *y*+1/2, −*z*+1/2; (iii) *x*−1, −*y*+3/2, *z*−1/2.
